# Supplementary material for: Impact of tourism on habitat use of black grouse (Tetrao tetrix) in an isolated population in northern Germany
Source: PLoS One. 2020 Sep 4;15(9):e0238660. doi: 10.1371/journal.pone.0238660 (PMC7473583; doi:10.1371/journal.pone.0238660)
Supplement: S1 Table — (DOCX) [file pone.0238660.s001.docx]

**S1 Table. Duration of visitor monitoring, numbers of recorded trigger events of all ten light barriers and assigned categorisation of visitor frequency.**

| **ID** | **Type of route** | **Start** | **End** | **Days** | **Number of triggers** | **Mean number of triggers per day** | **Category of visitor frequency** |
| --- | --- | --- | --- | --- | --- | --- | --- |
| 1 | public | 15.01.2015 | 06.05.2017 | 842 | 100,810 | 119.7 | very high (5) |
| 2 | public | 15.01.2015 | 13.05.2017 | 849 | 35,004 | 41.2 | high (4) |
| 3 | closed | 11.09.2015 | 13.10.2016 | 398 | 1,180 | 2.9 | low (1) |
| 4 | closed | 17.09.2015 | 13.10.2016 | 392 | 743 | 1.8 | low (1) |
| 5 | public | 17.09.2015 | 06.05.2017 | 597 | 23,321 | 39.0 | medium (3) |
| 6 | public | 25.09.2015 | 16.05.2017 | 599 | 18,430 | 30.7 | medium (3) |
| 7 | public | 01.10.2015 | 16.05.2017 | 593 | 133,896 | 225.7 | very high (5) |
| 8 | public | 01.10.2015 | 16.05.2017 | 593 | 15,610 | 26.3 | moderate (2) |
| 9 | public | 08.10.2015 | 16.05.2017 | 586 | 12,363 | 21.0 | moderate (2) |
| 10 | public | 15.10.2015 | 16.05.2017 | 579 | 31,457 | 54.3 | high (4) |
