# Supplementary material for: Impact of tourism on habitat use of black grouse (Tetrao tetrix) in an isolated population in northern Germany
Source: PLoS One. 2020 Sep 4;15(9):e0238660. doi: 10.1371/journal.pone.0238660 (PMC7473583; doi:10.1371/journal.pone.0238660)
Supplement: S2 Table — (DOCX) [file pone.0238660.s002.docx]

**S2 Table. Mean monthly numbers of light barrier triggers (years 2015-2017) and overnight stays in three municipalities (years 2009-2019) are highly correlated for all public routes except route ID 6.**

| **Route ID** | **2009** | **2010** | **2011** | **2012** | **2013** | **2014** | **2015** | **2016** | **2017** | **2018** | **2019** |
| --- | --- | --- | --- | --- | --- | --- | --- | --- | --- | --- | --- |
| 1 | 0.86 | 0.90 | 0.87 | 0.89 | 0.90 | 0.90 | 0.92 | 0.94 | 0.85 | 0.90 | 0.87 |
| 2 | 0.87 | 0.91 | 0.89 | 0.87 | 0.86 | 0.93 | 0.91 | 0.90 | 0.87 | 0.87 | 0.90 |
| 5 | 0.71 | 0.84 | 0.81 | 0.72 | 0.80 | 0.79 | 0.79 | 0.83 | 0.80 | 0.83 | 0.82 |
| 6 | 0.66 | 0.61 | 0.60 | 0.61 | 0.62 | 0.64 | 0.66 | 0.66 | 0.54 | 0.57 | 0.61 |
| 7 | 0.96 | 0.95 | 0.97 | 0.92 | 0.94 | 0.95 | 0.92 | 0.93 | 0.95 | 0.90 | 0.96 |
| 8 | 0.80 | 0.87 | 0.84 | 0.85 | 0.88 | 0.88 | 0.92 | 0.94 | 0.83 | 0.90 | 0.83 |
| 9 | 0.87 | 0.92 | 0.89 | 0.90 | 0.86 | 0.94 | 0.93 | 0.90 | 0.87 | 0.89 | 0.90 |
| 10 | 0.87 | 0.89 | 0.87 | 0.92 | 0.89 | 0.93 | 0.94 | 0.94 | 0.86 | 0.89 | 0.85 |
