# Supplementary figures and images for: Impact of tourism on habitat use of black grouse (Tetrao tetrix) in an isolated population in northern Germany
Source: PLoS One. 2020 Sep 4;15(9):e0238660. doi: 10.1371/journal.pone.0238660 (PMC7473583; doi:10.1371/journal.pone.0238660)

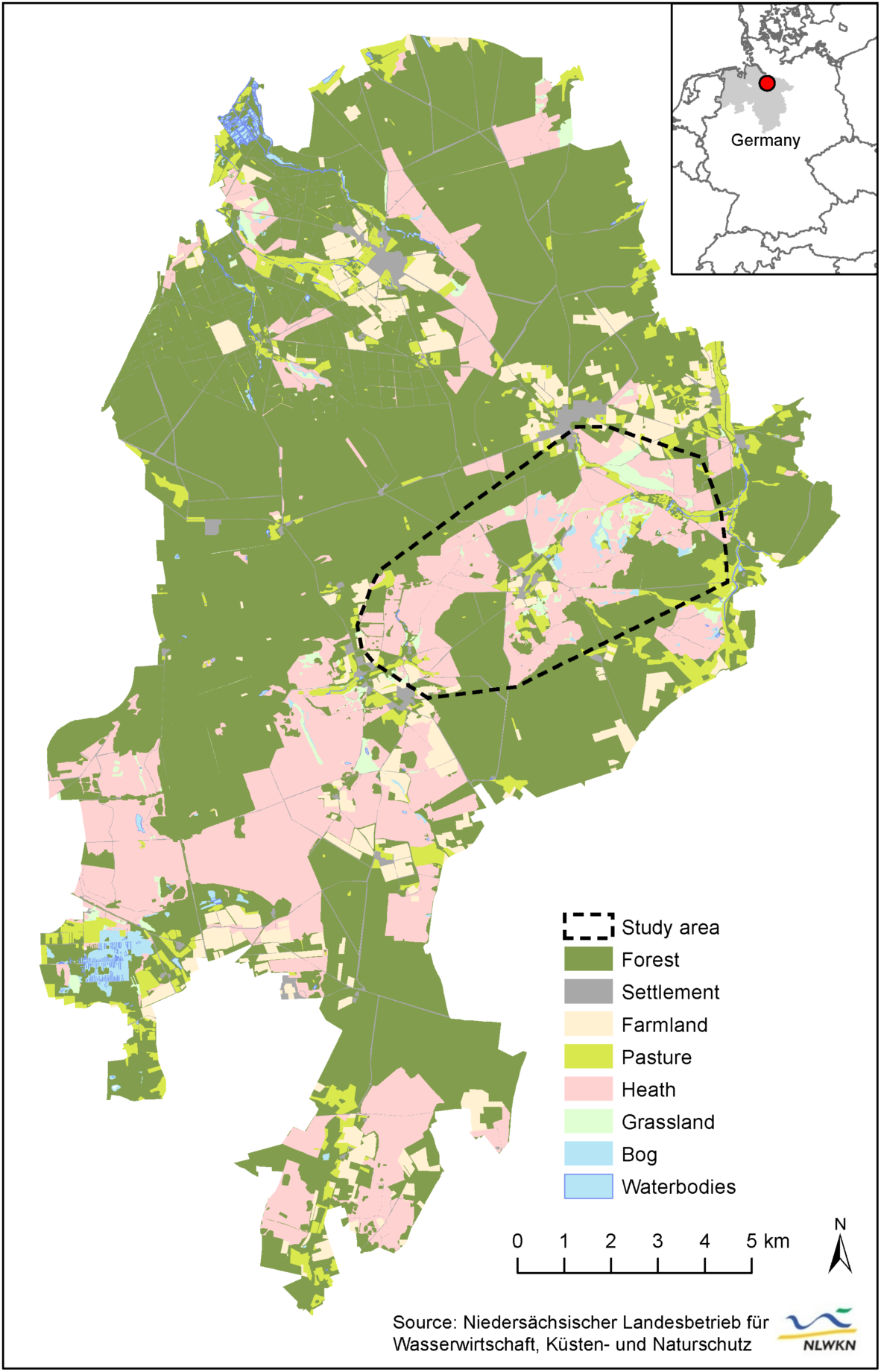

Supplement: S1 Fig — (TIF) [file pone.0238660.s003.tif]

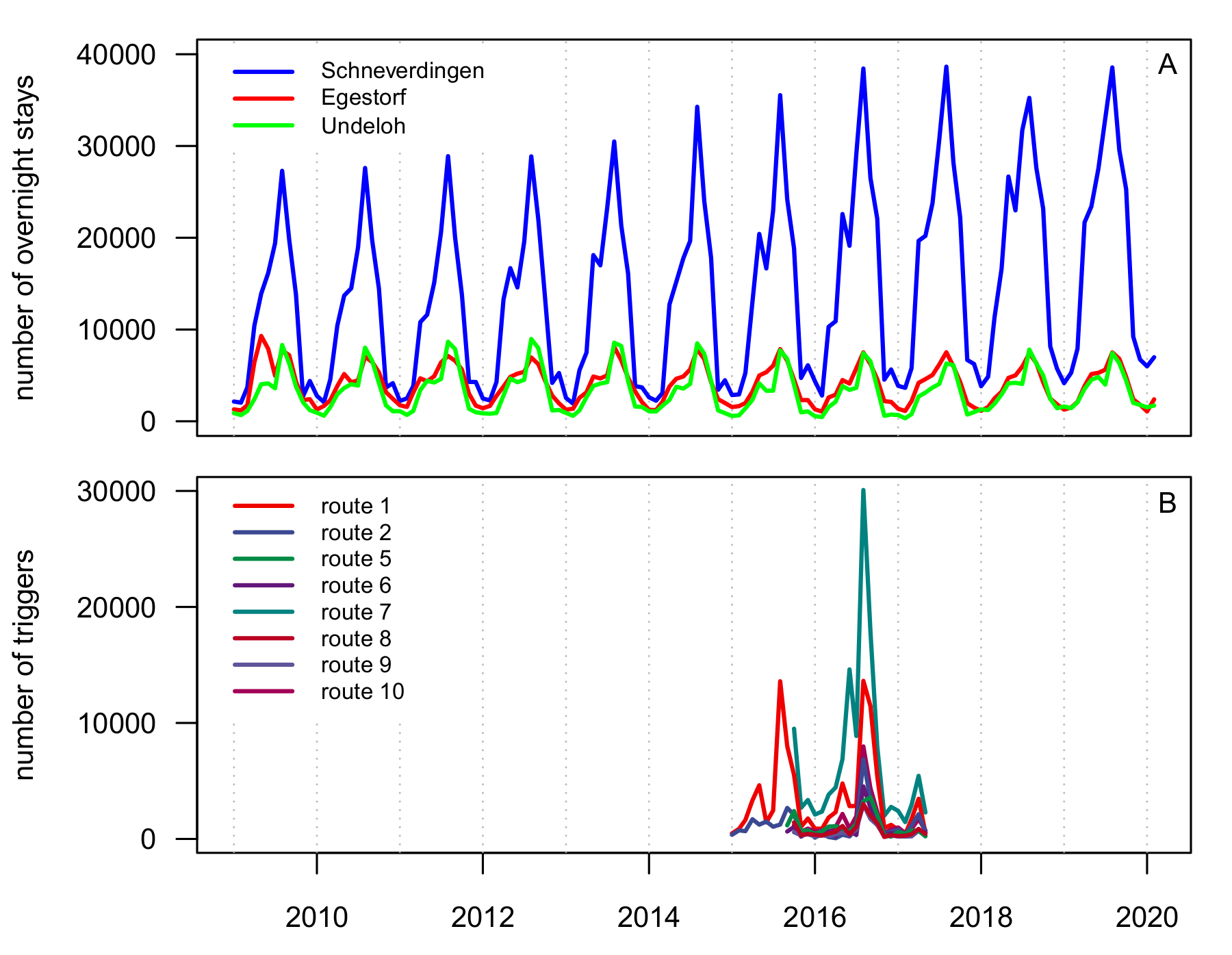

Supplement: S2 Fig — Comparison of numbers of guest overnight stays (A) and light barrier trigger events (B) per month. Overnight stays are monthly cumulated for three municipalities of the nature reserve Lüneburg Heath (Schneverdingen, Undeloh, Egestorf). Trigger events are monthly cumulated for each monitored public route. (TIF) [file pone.0238660.s004.tif]

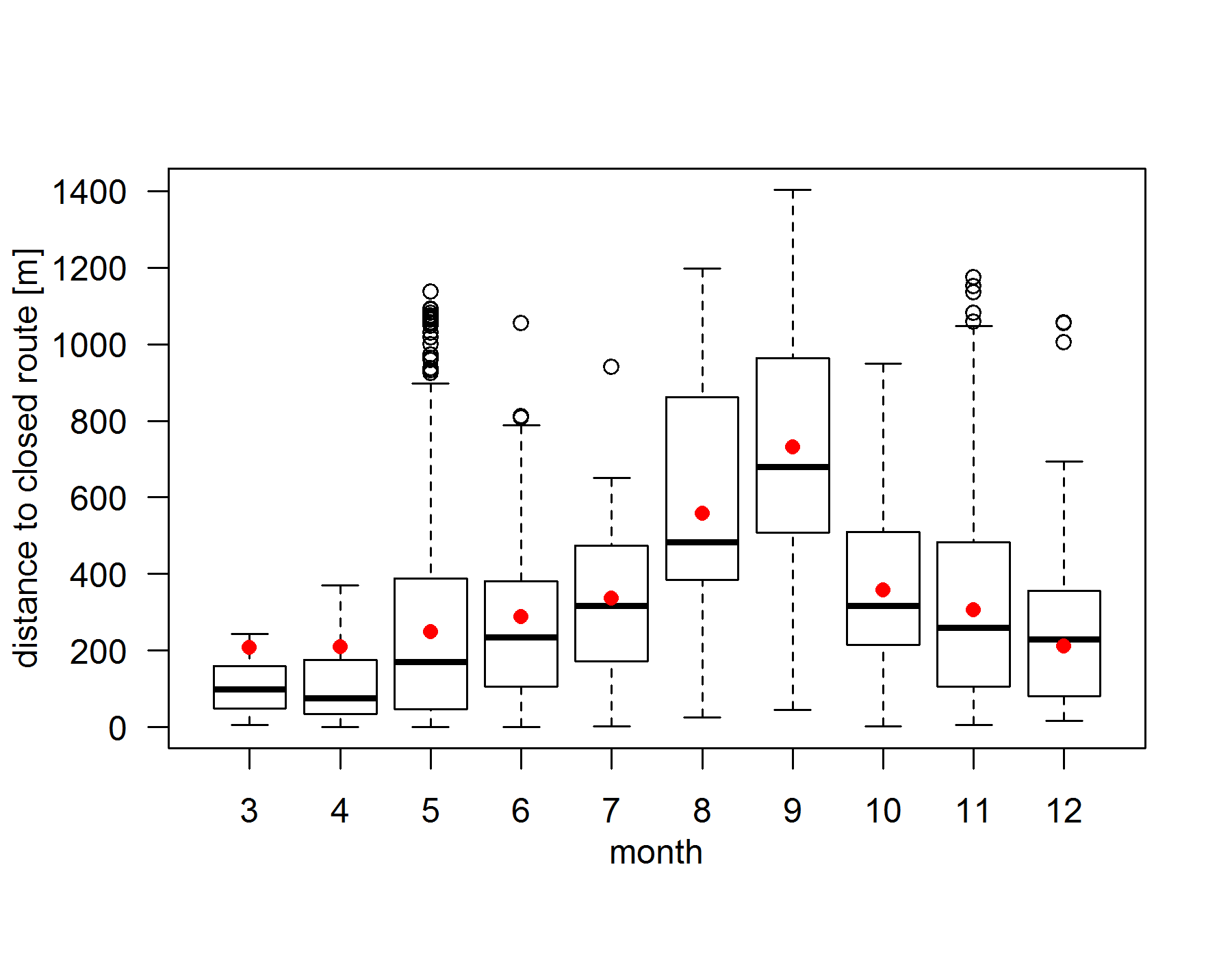

Supplement: S3 Fig — Distance distributions are visualised as boxplots by month; red marks show the linear mixed-model’s estimates of distance explained by monthly activity of visitors. Individuals were considered as random factors. (TIF) [file pone.0238660.s005.tif]
